# Clinical characteristics and treatment response of treatment requiring retinopathy of prematurity (ROP) in Big Premature Infants in Turkiye: BIG-ROP Study Group Report No 2 (BIG-ROP STUDY)

**DOI:** 10.1136/bmjophth-2024-002081

**Published:** 2025-06-05

**Authors:** Huseyin Baran Ozdemir, Sengul Ozdek, Zuhal Ozen Tunay, Sadik Etka Bayramoglu, Emine Alyamac Sukgen, Nur Kır, on behalf of BIG-ROP Study Group, Dilbade Yıldız Ekinci

**Affiliations:** 1 Department of Ophthalmology, Gazi University Faculty of Medicine, Ankara, Turkey; 2 Department of Ophthalmology, TOBB Ekonomi ve Teknoloji Üniversitesi Tip Fakültesi, Ankara, Turkey; 3 Department of Ophthalmology, İstanbul Kanuni Sultan Süleyman Eğitim ve Araştırma Hastanesi, Istanbul, Turkey; 4 Department of Ophthalmology, TC Saglik Bakanligi Adana Sehir Egitim ve Arastirma Hastanesi, Adana, Turkey; 5 Department of Ophthalmology, Istanbul University Istanbul Faculty of Medicine, Istanbul, Turkey

**Keywords:** Retina, Treatment Lasers, Treatment Surgery, Treatment other

## Abstract

**Objective:**

This study evaluated the clinical characteristics and treatment outcomes of bigger premature infants treated for retinopathy of prematurity (ROP).

**Methods:**

A retrospective, multicentre study analysed data from 33 ROP centres in Türkiye. Infants with gestational ages (GA) of 32–37 weeks and birth weights (BW) >1500 g who required ROP treatment were included. Patient demographics, clinical details, treatments, responses and complications were recorded. Descriptive statistics were calculated after excluding cases with missing or erroneous data.

**Results:**

The study included 365 eyes of 365 infants. The average GA at birth was 33±1 weeks, with a mean BW of 1896±316 g. Of these, 83.6% had type 1 ROP, and 16.4% had aggressive ROP (A-ROP). Treatment-requiring ROP (TR-ROP) occurred at an average postmenstrual age of 39.0±4.6 weeks. Among 170 infants with TR-ROP at their first exam, 81.2% were screened at 4 weeks postpartum. Reactivation of ROP was observed in 5.4% of the primary laser photocoagulation (LPC) group and 23.9% of the primary anti-vascular endothelial growth factor (VEGF) group (p<0.001). Reactivation and progression to stage 4–5 were more frequent in A-ROP cases (p=0.012; p=0.008). The need for additional treatment was significantly higher in cases of A-ROP, zone 1 disease or stage 4–5 disease (p<0.001). Anti-VEGF therapy demonstrated superior single-treatment success rates in A-ROP eyes compared with laser LPC (85.7% vs 60%, p=0.03). Infants requiring additional treatments also had higher rates of respiratory distress syndrome (RDS), maternal premature rupture of membranes (PROM) and non-ophthalmological surgical interventions (p<0.05).

**Conclusion:**

Bigger premature infants in low and middle-income countries should be screened earlier than 4 weeks after birth. A-ROP, zone 1 disease and stage 4–5 disease have higher reactivation risks. Primary anti-VEGF therapy was associated with a greater need for retreatment. Maternal PROM, RDS and surgical interventions also increase retreatment risk. Limitations include retrospective design and lack of smaller preterm comparisons, potentially limiting generalisability.

**WHAT IS ALREADY KNOWN ON THIS TOPIC**

- Retinopathy of prematurity (ROP) is a preventable cause of blindness in premature infants, with treatment-requiring ROP (TR-ROP) occurring more frequently in bigger and more mature preterm infants in low and middle-income countries. Previous studies highlighted that these infants were at risk of developing TR-ROP under suboptimal neonatal care, necessitating broader screening criteria to capture this at-risk population.

**WHAT THIS STUDY ADDS**

- This study demonstrates that TR-ROP in bigger and more mature preterm infants can be detected earlier with screening earlier than 4 weeks after birth. Laser photocoagulation achieves higher single-treatment success rates, while anti-vascular endothelial growth factor (VEGF) therapy is more effective for AROP in reducing retinal detachment. Key risk factors for increased severity of ROP include respiratory distress syndrome, prior surgical interventions and maternal premature membrane rupture.

**HOW THIS STUDY MIGHT AFFECT RESEARCH, PRACTICE OR POLICY**

- The findings support revising screening protocols in developing countries to initiate ROP examinations earlier than the 4th postpartum week for bigger preterms, emphasising tailored treatment approaches based on ROP type. Additionally, policies aimed at improving neonatal care quality and delaying elective surgeries in at-risk infants could mitigate severe ROP development.

## Introduction

 Retinopathy of prematurity (ROP) is a sight-threatening, vasoproliferative disease affecting the immature peripheral retina in premature infants. ROP-related blindness can be potentially avoidable with advanced neonatal care, effective screening/treatment programmes and correct/in-timing treatment. On the other hand, insufficient neonatal intensive care unit (NICU) conditions may even stimulate the development of ROP, which is the case in low and middle-income countries (LMICs) and treatment-requiring ROP (TR-ROP) may be observed even in bigger premature infants.^1^ That is why we suggest that those infants experiencing suboptimal neonatal care need to be screened with broader screening criteria.^2^

The Republic of Turkiye Ministry of Health requires screening preterm infants with gestational age (GA) ≤32 weeks or birth weight (BW) ≤1500 g or infants with a BW >1500 g and GA >32 weeks who had cardiopulmonary resuscitation or a defined extra risk factor for ROP by the paediatrician. A previous multicentre prospective study in Turkiye showed that the rates of severe ROP were higher in non-university NICUs, especially in private hospitals.^3^ The study also revealed that more mature infants are at risk of developing severe ROP in Turkiye and indicated that screening criteria should be wide enough to detect the majority of infants developing TR-ROP. Following these publications, Turkiye ROP Screening Guideline written by ROP commission of the Turkish Ophthalmological Association (TOA) and Turkish Neonatology Society has recently been revised in 2021 to enlarge the screening limits to involve the preterm infants with a GA ≤34 weeks or BW ≤1700 g or infants with a BW >1700 g and GA >34 week with a defined extra risk factor for ROP by paediatrician are advised for screening.^4^

The first report of our study group previously reported that more mature and heavier preterm infants might develop TR-ROP mainly when the ideal NICU conditions cannot be provided, and revision for screening criteria for ROP in Turkiye was needed.^2^ This study specifically investigates TR-ROP in a less-studied subgroup (GA: 32–37 weeks; BW>1500 g). While smaller and less mature preterm infants have been extensively studied, bigger preterms remain under-represented in the literature. The physiological and clinical differences between large and small preterms in the development of TR-ROP are due to differences in retinal vascularisation and oxygen regulation. Bigger preterms often experience TR-ROP at earlier period after birth. In this study, as the second report of the same group, we aimed to evaluate the treatment choices, responses, complications and relationship between treatment responses and clinical characteristics in preterm infants with a GA >32 week and BW >1500 g who developed TR-ROP.

## Methods

This non-randomised, retrospective, multicentre study was conducted by the TOA ROP commission to evaluate the demographic and clinical features of preterm infants with a GA >32 week and BW >1500 g who developed TR-ROP. Members of the TOA who wish to contribute were requested to fill out the study-specific data entry forms for each patient treated for TR- ROP.^5^ Data were collected from January 2003 to December 2020 across 33 centres, ensuring all eligible consecutive cases treated by participating ophthalmologists were included. As a result, the study organisers (SO and HBO) received data from 713 eyes of 376 patients. Patients or the public were not involved in the design, or conduct, or reporting or dissemination plans of our research

The study included infants with GA between 32 weeks and 37 weeks and BW ≥1500 g who developed TR-ROP retrospectively. Inclusion criteria were GA 32–37 weeks and BW >1500 g, excluding infants below these thresholds or those not meeting treatment criteria. Clinical and demographic data of the patient cohort were reported in the previous publication from our study group.^2^ The present study focused more on the initial treatment choices such as laser photocoagulation (LPC), intravitreal anti-VEGF (anti-VEGF) or vitreoretinal surgery (VRS), treatment responses, complications, retreatment rates and the relationship between retreatment and clinical characteristics reported before were collected. Descriptive statistics excluded erroneous data. Cases with incomplete neonatal assessment but consistent treatment criteria were included to minimise bias.

Infants were treated per contemporary clinical practices during the study period. LPC was preferred for zone 2 plus disease, while anti-VEGF was administered for A-ROP and zone 1 involvement based on published evidence and expert consensus at the time (Mintz-Hittner *et al*; Stahl *et al*).^6 7^ For posterior zone 2 disease, LPC or anti-VEGF treatment was administered according to clinician decision. VRS was performed for stage 4 and 5 ROP.

Statistical analyses were performed using SPSS V.22.0. The normal distribution of the continuous variables was tested using the Kolmogorov-Smirnov test and the homogeneity of variance was tested using Levene’s test. Descriptive statistics as frequencies, percentages, mean (± SD) and median values (minimum, maximum) were first calculated for all variables of interest. Since the quantitative variables were unsuitable for the normal distribution, they were analysed by the Mann-Whitney U test. Pearson’s χ^2^, Fisher’s exact, likelihood ratio and McNemar’s tests were used to compare categorical data. Multiple logistic regression analyses were conducted using a prospective selection approach, specifically forward stepwise selection, to identify key risk factors for additional treatment. Variables (eg, GA at birth, pretreatment zone, stage and treatment type) were entered iteratively based on their statistical significance (p<0.05), starting with the most significant predictor, until no further variables improved the model fit, as assessed by the likelihood ratio test. Statistical analyses did not apply Bonferroni correction for multiple comparisons, given the exploratory nature of the study, though p values <0.05 were considered.

## Results

Data of 376 infants treated between January 2003 and December 2020 from 33 reference centres for ROP, 16 university hospitals, 15 state hospitals and 2 private hospitals were received. Eleven patients were excluded due to insufficient data or duplication, and 365 patients were included in the study. The 33 participating centres had ophthalmology units for ROP screening. All centres performed LPC and anti-VEGF treatments; only one centre performed VRS. The mean GA of infants was 33±1 (32^1^ – 37) weeks, and the mean BW was 1896±316 g (1505–3760 g).

### Primary treatment results

The first examination was performed on time (postpartum week 4–5) in 293 (80.3%) infants. 170 patients (46.7%) had TR-ROP at first examination (online supplemental figure 1). Sixty infants (16.4%) had A-ROP, and 305 (83.6%) had type 1 ROP. TR-ROP occurred at a mean postmenstrual age (PMA) of 39.0±4.6 weeks. The most common pretreatment zone was zone 2 (73.7%). Two hundred twenty-four patients (61.4%) received LPC, 134 (36.7%) patients underwent anti-VEGF treatment (most commonly bevacizumab, 70.2%), and seven (1.9%) patients underwent VRS as an initial treatment. There was no difference between laser and anti-VEGF groups with respect to GA (33.03±1.05 week vs 33.00±1.01 week, p=0.813) and BW (1909.57±330.18 g vs 1868.52±288.27 g, p=0.234). However, zone 2 disease and stage 3 ROP were more prevalent in the LPC group than in the anti-VEGF group, with rates of 80.8% (181 patients) versus 61.2% (82 patients) for zone 2 and 91.9% (206 patients) versus 61.9% (83 patients) for stage 3 (p<0.001 for both). Zone 1 disease and A-ROP were more common in the anti-VEGF group than LPC group, with 38.1% (51 patients) versus 5.8% (13 patients) for zone 1 and 36.6% (49 patients) versus 4.5% (10 patients) for A-ROP (p<0.001 for both). Type 1 ROP was predominant in the LPC group (95.5%, 214 patients) compared with 63.4% (85 patients) in the anti-VEGF group. Plus disease was present in 87.9% (197 patients) of LPC cases and 98.5% (132 patients) of anti-VEGF cases (p<0.001).

Of 293 infants screened at 4–5 weeks postpartum, 138 (47.1%) had TR-ROP at their first examination. TR-ROP at first examination group had a significantly higher mean BW (1932.86±349.53 g) as compared with the others (1852.21±279.58 g, p=0.030). GA showed no significant difference p=0.561, with means of 33.05±1.07 weeks in TR-ROP group and 32.98±0.99 weeks in the non-treated group. NICU-level data revealed a significant difference (p=0.016), with TR-ROP at the first examination being less frequent in level 3B–4 NICUs (9.4% in TR-ROP at first examination vs 20.1% in the others) than in level 1–2 (22.5% vs 14.3%, respectively) and level 3A (68.1% vs 65.6%, respectively). Other prematurity-related complications and maternal factors showed no significant difference (p>0.05 for all).

Three hundred and twenty (87.7%) infants required no additional treatment, while 45 (12.3%) infants required additional treatments (table 1). The rate of need for additional treatment was 5.4% in the LPC-treated eyes and 23.9% in the anti-VEGF-treated eyes (table 2), which was significantly higher in anti-VEGF group as compared with LPC group (p<0.0001). Of the 32 patients who needed additional treatment following anti-VEGF, 5 (15.6%) received second anti-VEGF injection, 16 (50.0%) received LPC because of reactivation or progression as a rescue treatment. Eleven of 32 (34.4%) patients received LPC for ablation of persistent peripheral avascular retina (PAR) at a median PMA of 96 weeks (IQR: 60–141 weeks), considered prophylactic rather than retreatment for active disease recurrence, and thus excluded from the retreatment group in subsequent analyses unless specified. The need for additional treatment was still significantly higher in the Anti-VEGF group even after excluding patients who underwent LPC for PAR (p=0.002). Reactivation occurred in 14 of 94 infants (14.9%) injected with bevacizumab, 3 of 18 infants (16.7%) injected with ranibizumab and 4 of 22 infants (18.2%) injected with aflibercept. There was no significant difference in ROP reactivation between anti-VEGF types (p=0.854). VRS as the initial treatment achieved complete recovery (no recurrence of active ROP or need for further treatment) in six of seven patients (85.7%) with a single surgery. Primary treatment results are detailed in table 2.

**Table 1:** Clinical characteristics related to ROP (n=365)

First examination week	Patients, n (%)
Week 4–5	293 (80.3%)
Week 6–7	72 (19.7%)
Presence of any ROP at first exam	328 (89.9%)
TR-ROP diagnosis, wk	PP 6.7±4.7 wk (5–7 wk) PMA 39.0±4.6 wk (38–41 wk)
TR-ROP at first exam	170 (46.7%)
TR-ROP at first exam at fourth pp wk	138 of 170 (81.2%)
TR-ROP at 1st exam at > 4th pp wk	32 of 170 (18.8%)
ROP type	
A-ROP	60 (16.4%)
Type 1	305 (83.6%)
Pre-treatment zone	
Zone 1	65 (17.8%)
Zone 2	269 (73.7%)
Zone 3	31 (8.5%)
Time of treatment	PP 6 wk (5–7 wk) PMA 39 wk (38–41 wk)
Initial treatment choice	
LPC	224 (61.4%)
Anti-VEGF	134 (36.7%)
VRS	7 (1.9%)
Need for additional treatment	
No	320 (87.7%)
Yes	45 (12.3%)

Quantitative data are summarised as median (first quarter–third quarter), and qualitative data are summarised as frequency (percentage).

LPC, laser photocoagulation; PMA, post-menstrual age; PP, post-partum; ROP, retinopathy of prematurity; TR-ROP, treatment-requiring retinopathy of prematurity; VEGF, vascular endothelial growth factor; VRS, vitreoretinal surgery; wk, week.

**Table 2:** Treatment outcomes

**LPC**	**224 of 365 patients (61.4%)**
LPC time (median)	PP 7 wk (5–9 wk) PMA 40 wk. (38–42 wk)
*Results after LPC (n=224*)	
Complete recovery (patients)	212 (94.6%)
Secondary treatment (patients)	12 (5.4%)
Second LPC for skip areas	9 (4.1%)
VRS (progression to stage 4)	6 (3 after the first LPC, 3 after the second LPC) (2.6%)
Additional treatment time after LPC, wk after first treatment	2 wk (IQR: 1–2.75 wk.)
**Anti-VEGF injection**	**134 of 365 patients (36.6%)**
Anti-VEGF type	
Bevacizumab	94 (70.2%)
Ranibizumab	18 (13.4%)
Aflibercept	22 (16.4%)
Anti-VEGF treatment time (median)	PP 5 wk (4–6 wk) PMA 38 wk (37–39 wk)
*Results after anti-VEGF injection (n=134*)	
Complete recovery (patients)	102 (76.1%)
Need for additional treatment (patients)	32 (23.9%)
Second anti-VEGF	5 (3.7%)
Anti-VEGF for ROP reactivation	3 (2.2%)
Anti-VEGF for non-regression	2 (1.5%)
Second anti-VEGF re-treatment time (median)	1 wk (IQR: 1–2 wk)
LPC	27 (20.2%)
LPC for ROP reactivation	13 (9.7%)
LPC for non-regression	1 (0.8%)
LPC for progression to stage 4	2 (1.5%)
LPC for peripheral avascular retina (PAR)*	11 (8.2%)
LPC retreatment time,	16 wk (IQR: 9–36 wk)
LPC retreatment time for PAR (median)	96 wk (IQR: 60–141 wk)
VRS as the third treatment (patients)	3 of 134 (2.2%)

Quantitative data are summarised as median (first quarter–third quarter), and qualitative data as frequency (percentage).

* LPC for PAR (11 patients) was prophylactic and excluded from retreatment analyses unless specified.

LPC, laser photocoagulation; LSV, lens-sparing vitrectomy; LV, lensectomy-vitrectomy; PMA, Post-menstrual age; PP, post-partum; ROP, retinopathy of prematurity; TRD, tractional retinal detachment; VEGF, vascular endothelial growth factor; VRS, vitreoretinal surgery; wk, week.

The median PMA at the time of the initial treatment was 39 (38–41) weeks, which was 40 weeks (IQR 38–42 week) in LPC group, 38 weeks (IQR 37–39 week) in the anti-VEGF group and 44 weeks (IQR 39–68 weeks) in the VRS group. The anti-VEGF injection group received treatment significantly earlier than the other groups (p<0.0001). Anti-VEGF injection was preferred mostly in A-ROP patients and zone 1 disease (p<0.0001).

### Results according to the type of ROP

There were 305 patients with type 1 ROP and 60 patients with A-ROP. Among type 1 ROP, 214 patients (70.2%) received LPC and 85 (27.9%) received anti-VEGF injection. Among the 60 patients with A-ROP, 10 patients (16.7%) were treated with LPC and 49 (81.7%) were treated with anti-VEGF injection as the primary treatment.

Rates of retreatment (excluding LPC for PAR) and progression to stage 4–5 were listed in table 3. The risk of ROP reactivation was higher in A-ROP patients, even after excluding patients who underwent LPC due to PAR (p=0.012). In type 1 ROP patients, ROP reactivation was statistically higher in those treated with anti-VEGF (p<0.0001). The need of VRS (progression to stage 4–5) was statistically significantly higher in A-ROP patients than in type 1 ROP patients (p=0.008). Progression to stage 4–5 was more prevalent in the primarily LPC-treated A-ROP patients than primarily anti-VEGF-injected A-ROP patients (p=0.03).

**Table 3:** Rates of ROP reactivation and progression to stage 4 and 5 ROP among types of ROP

	Type 1 ROP (n: 299)	A-ROP (n: 59)	P value
ROP reactivation	22 (7.4%)	11 (18.6%)	0.012
LPC (n: 224)	8 of 214 (3.7%)	4 of 10 (40%)	0.001
Anti-VEGF (n:134)	14 of 85 (16.5%)	7 of 49 (14.3%)	0.675
P value	<0.0001	0.079	
Progression to stage 4&5	4 (1.3%)	5 (8.5%)	0.008
LPC (n: 224)	3 of 214 (1.4%)	3 of 10 (30%)	0.001
Anti-VEGF (n:134)	1 of 85 (1.2%)	2 of 49 (4.1%)	0.554
P value	0.878	0.03	

P values are unadjusted for multiple comparisons; interpret with caution due to the risk of type I error.

A-ROP, aggressive ROP; LPC, laser photocoagulation; PAR, persistent avascular retina; ROP, retinopathy of prematurity; VEGF, vascular endothelial growth factor.

### Secondary treatment results

The retreatment rate was statistically significantly higher in the anti-VEGF injection group (32 of 134 patients, 23.9%) than the LPC group (12 of 224 patients, 5.4%) and the VRS group (one of seven patients, 14.3%). There was no statistically significant difference with respect to the first examination week, presence of ROP at the first examination and time to TR-ROP development in patients with or without additional treatment. The need for additional treatment was higher in A-ROP (p<0.001), zone 1 disease (p<0.001) and stage 4 and 5 disease (p<0.001).

The GA at birth was higher in the group requiring additional treatment (p=0.015, 33 vs 34 wk). The frequency of ROP retreatment was significantly higher in patients who underwent non-ophthalmic surgical interventions (eg, abdominal or thoracic procedures unrelated to ocular conditions) (p=0.034). The frequency of surgical treatment was found to be statistically significantly higher in babies whose mothers had PMR and had RDS (p<0.05). There was no statistically significant difference between the groups regarding BW, intensive care unit characteristics, other diseases, maternal age and prenatal characteristics in terms of retreatment.

After primary treatment, 36 of 45 (80%) patients received LPC as a second (rescue) treatment (table 2). Twenty-seven (75%) of 36 patients who underwent LPC as a secondary treatment have primarily been treated with intravitreal anti-VEGF injection and the rest of the patients have initially been treated with LPC (25%). The most common indications for LPC after primary intravitreal anti-VEGF were reactivation of ROP (13 patients, 48.1%) and PAR (11 patients, 40.7%). Five (3.7%) of the anti-VEGF-treated patients needed a second anti-VEGF treatment; 3 (2.2%) for reactivation of ROP and 2 (1.5%) for non-regression in plus disease. VRS was needed in a total of six (2.6%) patients, all of whom have been primarily treated with LPC.

### VRS results

A total of 16 (4.4%) patients underwent VRS. Seven (43.8%) of these had the VRS as the primary treatment and the others as a secondary treatment; six following LPC (37.5%) and three after anti-VEGF (18.8%) as the primary treatment (table 3). 14 patients (87.5%) were treated with single surgery and 2 patients (12.5%) needed a second VRS. Lens-sparing vitrectomy (LSV) was performed in 10 (62.5%) patients, while LV was performed in 6 (37.5%) patients (table 4). The final anatomical success was achieved in all patients (100%) who underwent LSV and in 50.0% who underwent LV (p=0.036).

**Table 4:** Treatment outcomes of patients who underwent VRS (n: 16)

	Primary VRS (n: 7)	VRS after LPC (n: 6)	VRS after anti-VEGF (n: 3)
Stage 4A ROP	0	5	2
Time for VRS (PP wk)	–	6 (6 – 22)	10
LSV	–	5	2
Final anatomical success	–	5 (100%)	2 (100%)
Stage 4B ROP	2	0	1
Time for VRS (PP wk)	6 (5-7)		9
LSV	1	–	1
Final anatomical success	1 (100%)	–	1 (100%)
LV	1	–	–
Final anatomical success	1 (100%)	–	–
Stage 5 ROP	5	1	–
Time for VRS (PP wk)	23.5 (4 – 77)	7	–
LSV	1	0	–
Final anatomical success	1 (100%)	–	–
LV	4	1	–
Final anatomical success	2 (50%)	0 (0%)	–

LPC, laser photocoagulation; LSV, lens-sparing vitrectomy; LV, lensectomy-vitrectomy; PMA, postmenstrual age; PP, post-partum; VEGF, vascular endothelial growth factor; VRS, vitreoretinal surgery; wk, week.

### Risk factors for ROP reactivation

Multiple logistic regression analysis was performed to determine essential risk factors for additional treatment. After the correction was made according to the GA at birth, the pretreatment zone, the pretreatment stage and type of treatment were determined as significant risk factors for additional treatment. Zone 1 eyes had a 2.34-fold higher need for additional treatment than others (OR: 2339, 95% CI 1100 to 4976, p=0.027). Cases with pretreatment stages 4 and 5 had a 7.7-fold higher risk for additional treatment than others (OR: 7,696, 95% CI 2703 to 21 911, p<0001). Eyes treated with anti-VEGF had a 4.6-fold higher risk of additional treatment than others (OR: 4587, 95% CI 2110 to 9975, p<0001) (table 5).

**Table 5:** Risk factors for need of additional treatment, multiple logistic regression analysis

	β coefficient	SE	P value	OR	OR to 95.0% GA	
GA at birth	0.368	0.147	**0.012**	1.445	1.084	1.926
Zone 1 disease	0.850	0.385	**0.027**	2.339	1.100	4.976
Pre-treatment stages	2.041	0.534	**<0001**	7.696	2.703	21.911
Anti-VEGF therapy	1.523	0.396	**<0001**	4.587	2.110	9.975
Constant	−15.360	4.919	**0.002**			

Dependent variable: the need for additional treatment; Nagelkerke R2=0.241; accurate prediction rate=87.67%.

Bold values indicate statistical significance at the p<0.05 level.

GA, gestational age; VEGF, vascular endothelial growth factor.

At the final examination (median 16 months (6–34 months)), 352 (98.9%) infants had attached retina. One patient developed phthisis bulbi (0.2%), two patients developed partial retinal detachment (0.4%), and one patient developed total retinal detachment (0.2%) during follow-up.

## Discussion

The present study evaluated clinical characteristics and treatment responses of premature infants bigger than 32 weeks and heavier than 1500 g who developed severe ROP, which remains a serious health problem in LMICs. In the literature, there are few studies with limited number of patients on the results of these bigger premature infants. This study stands out for its significant contribution to the literature due to its larger sample size.

The most striking finding was that almost 90% of infants had ROP of any stage and almost half had TR-ROP at the first examination. Although our inclusion criteria targeted bigger preterm infants (GA 32–37 weeks, BW >1500 g) with TR-ROP, this highlights the vulnerability of this subgroup in suboptimal care settings. Of the 170 infants with TR-ROP at first examination, 32 (18.8%) were screened later than 4 weeks postpartum, while 138 (81.2%) were examined on time at the fourth postpartum week. This is important because TR-ROP develops mostly at PMA of 34–38 weeks almost independent of GA at birth (younger or older).^7^,^9^ In this study, it has been shown that TR-ROP develops mostly at these weeks of PMA, which is only a few weeks after birth in these older preterms. This suggests that the standard screening at fourth week postpartum may be too late for this subgroup, supporting a recommendation for screening at earlier than 4 weeks postpartum. A study from China has reported that smaller prematures (born at a GA of <28 weeks) were treated at a PMA of 35 weeks, and those born at 32–33 weeks were treated at a GA of 38 weeks, underlining that duration between GA and treatment time decreases as GA increased, which supports our finding.^9^ These results suggest that, earlier examination (eg, at 3 weeks) in more mature preterm infants is of great importance to detect potential severe ROP, which is not expected frequently in such infants. Although the proposed postpartum third week screening cut-off lacks direct statistical validation in this retrospective study, it comes from the observation that TR-ROP occurred at a mean PMA of 39.0±4.6 weeks, often within 6–7 weeks postpartum (table 1). For infants with GA >32 weeks, delaying screening to 4 weeks (PMA ~36–37 weeks) may miss some of the cases developing TR-ROP as early as 34 weeks PMA, as reported in the literature.^9^ A more comprehensive prospective study for earlier screening of such bigger preterm infants (GA 32–37 weeks and BW >1500 g) would better elucidate incidence, progression, and optimal screening timing.

LPC has been considered as the golden standard in the treatment of ROP in the previous years.^10^ However, recent evidence suggests that anti-VEGF treatment is also very effective, especially in Zone 1 disease.^11^,^14^ In the BEAT-ROP study, bevacizumab was more successful than LPC in zone 1 disease.^6^ Similarly, in RAINBOW, ranibizumab was more successful than LPC both in zone 1 and zone 2 disease.^7^ In support of these findings, in FIREFLEYE, aflibercept was superior to LPC in all types of ROP including A-ROP.^8^ All of these studies were on infants younger than 32 weeks. In the present study, in general, complete recovery could be obtained more after LPC (94.6%) than after anti-VEGF therapy (76.1%). However, in the subgroup of A-ROP, anti-VEGF therapy (85.7%) was superior to laser (60%) in getting complete recovery with single treatment for acute disease. Consistent with the literature,^15 16^ we observed a higher ROP reactivation rate with anti-VEGF (23.9%) compared with LPC (5.4%) as well as increased retreatment needs in A-ROP, zone 1 and advanced-stage disease. This study is valuable in that, it is the first report of the success rates of laser and anti-VEGF treatment in bigger preterms (GA >32 wk or BW >1500 g) with appropriate indications.

In the present study, tractional RD developed in 2.6% of patients who underwent LPC and 2.2% of those who received anti-VEGF injections. Progression to retinal detachment was significantly higher in A-ROP patients (8.5%) compared with type 1 ROP patients (1.5%). Notably, among A-ROP patients, 30% of those treated with LPC developed tractional retinal detachment (RD) compared with only 4.1% of those treated with anti-VEGF. Barry *et al* reported a significantly higher incidence of tractional RD after LPC (7.9%) compared with anti-VEGF (0%) in eyes with type 1 ROP treated before 36 weeks PMA. However, the risk was similar in eyes treated at or after 36 weeks PMA.^17^ All patients in our study were treated at or after 36 weeks of PMA and progression to tractional RD in type 1 ROP patients was similar between the treatment groups, in agreement with the study by Barry *et al.* The present study showing better anatomical results with less tractional RD development with anti-VEGF therapy in bigger premature infants with A-ROP indicates that these bigger A-ROP cases seem to benefit from anti-VEGF as the first choice, though this finding requires validation in broader populations.

Beyond low BW, low GA and oxygen therapy, it is well known that factors like multiple pregnancies, PMR, anaemia, pulmonary issues, ventilation and malnutrition raise ROP risk and severity.^18^ Cao *et al* tied reactivation to ROP severity.^19^ Our study found increased retreatment needs following non-ophthalmic surgeries, due to worsening of ROP secondary to systemic decline, aligning with the study of Owen *et al.*^20^ We support delaying elective surgeries until a PMA beyond ROP risk.^20^ PMR and RDS were associated with higher retreatment rates (p<0.05) in our study, potentially increasing the risk of progression to retinal detachment requiring VRS, possibly via inflammatory-oxidative stress.^21^,^23^ Infants with these risk factors need close monitoring for aggressive ROP.

There is limited data in the literature on treatment selection after ROP reactivation, which is often based on clinical experience. In our study, none of the eyes treated with LPC received anti-VEGF for reactivation, while 23.8% of the infants initially treated with anti-VEGF received a second injection, and 76.2% underwent LPC as rescue therapy. This highlights the preference for LPC over anti-VEGF as a secondary treatment for ROP in bigger preterms in Türkiye.

PAR is commonly observed after anti-VEGF treatment for posterior disease, with a 75% rate in our previous study.^24^ While the meta-analysis of RCTs^25^ reported a 30.6% LPC rate for PAR, this rate was only 8.2% in our study, likely due to its retrospective design and the lack of consensus on PAR treatment, which is often clinician dependent.

VRS is a therapeutic option for advanced ROP cases progressing to stages 4 and 5 ROP. The success rate of VRS for stage 4A ROP has been reported to be as high as 90%.^26^ Stage 4A ROP cases benefit more from VRS, and good anatomical outcomes can be provided in selected stage 5A ROP and cicatricial cases.^27^ Ozsaygili *et al* reported that 17.4% of stage 4A/B patients underwent a second surgery in their cohort.^26^ In our study, the rate of second surgery was 12.5%. Overall success rate was quite satisfactory in the presented series; retinal reattachment was achieved in 81.3% of the patients, which was higher in stage 4 ROP.

Genetic factors, such as those in familial exudative vitreoretinopathy, may contribute to TR-ROP in bigger preterms, warranting future genetic studies.^28^ Another important factor which predisposes bigger preterms to develop severe ROP is the poor neonatal care in under-equipped NICUs, leading to ‘oxygen-induced retinopathy’.^18 29^ When we further analyse the group with TR-ROP at the first examination, the BW was significantly higher and the NICU level was lower in this group. This may imply that TR-ROP in these infants primarily reflects disparities in neonatal care rather than traditional risk profiles, emphasising the need for enhanced screening and standardised care practices for bigger preterm infants across NICU levels. Unmonitored oxygen levels and poor health management can trigger an aggressive, atypical ROP, potentially explaining A-ROP in bigger preterms in countries like Türkiye. Recent studies stress tailoring ROP screening to local needs, especially where neonatal care varies.^30 31^ In Mexico, India, and Korea, ROP in older preterms (GA >30 weeks, BW >1500 g) is often missed under standard guidelines.^32^,^34^ Based on our data and the guideline by Turkish Neonatology Society/TOA 2021, we recommend Turkish Ministry of Health to revise ROP screening legislation to include neonates with a GA≤34 weeks or BW ≤1700 g and to start screening bigger preterms (with GA >32 weeks) at earlier than postpartum 4 weeks especially in poor-care settings.^35^ This broader inclusion criteria will help to identify high-risk cases earlier, enabling timely intervention and reducing blindness due to ROP.

This study has some limitations because of its retrospective and multicentre nature. In addition, since only prematures with a GA >32 week and BW>1500 g were included in the study, we could not compare the treatment responses between our cohort and smaller prematures. Another limitation is the lack of detailed data related to NICU stay and final ophthalmological examination of some of the patients. On the other hand, this study has the highest number of patients in the literature evaluating treatment responses in bigger prematures. Our findings encourage the screening of these bigger prematures at earlier than fourth postpartum week since TR-ROP is detected in a very high percentage of them at the first examination, especially in low NICU conditions. Although the single treatment success rate was higher in eyes treated with LPC, reactivation of ROP and progression to RD were more in A-ROP treated with LPC. RDS, any surgical intervention other than ophthalmic intervention, and the history of PMR in the mother increase the severity of ROP, so all the elective surgeries are suggested to be postponed avoiding severe ROP development. Although our study lacks longitudinal data on ROP incidence and timing across multiple screening points, screening before 4 weeks postpartum may be warranted in these bigger preterm infants, given the high TR-ROP rate observed at the first examination (46.7%), potentially driven by suboptimal NICU conditions, oxygen-induced retinopathy or genetic factors. Future prospective studies with serial examinations are needed to validate this recommendation statistically.

In conclusion, our study provides valuable insights into the clinical characteristics and treatment responses of bigger premature infants with severe ROP. High anatomical outcomes can be provided. Patients with A-ROP should be treated with intravitreal anti-VEGF injection primarily. These infants should be examined earlier than fourth postpartum week to avoid having stage 4–5 disease at the first examination.

## Supplementary material

10.1136/bmjophth-2024-002081 online supplemental file 1

## Data Availability

Data are available upon reasonable request.
