# Supplementary figures and images for: Clinical characteristics and treatment response of treatment requiring retinopathy of prematurity (ROP) in Big Premature Infants in Turkiye: BIG-ROP Study Group Report No 2 (BIG-ROP STUDY)
Source: BMJ Open Ophthalmol. 2025 Jun 5;10(1):e002081. doi: 10.1136/bmjophth-2024-002081 (PMC12142119; doi:10.1136/bmjophth-2024-002081)

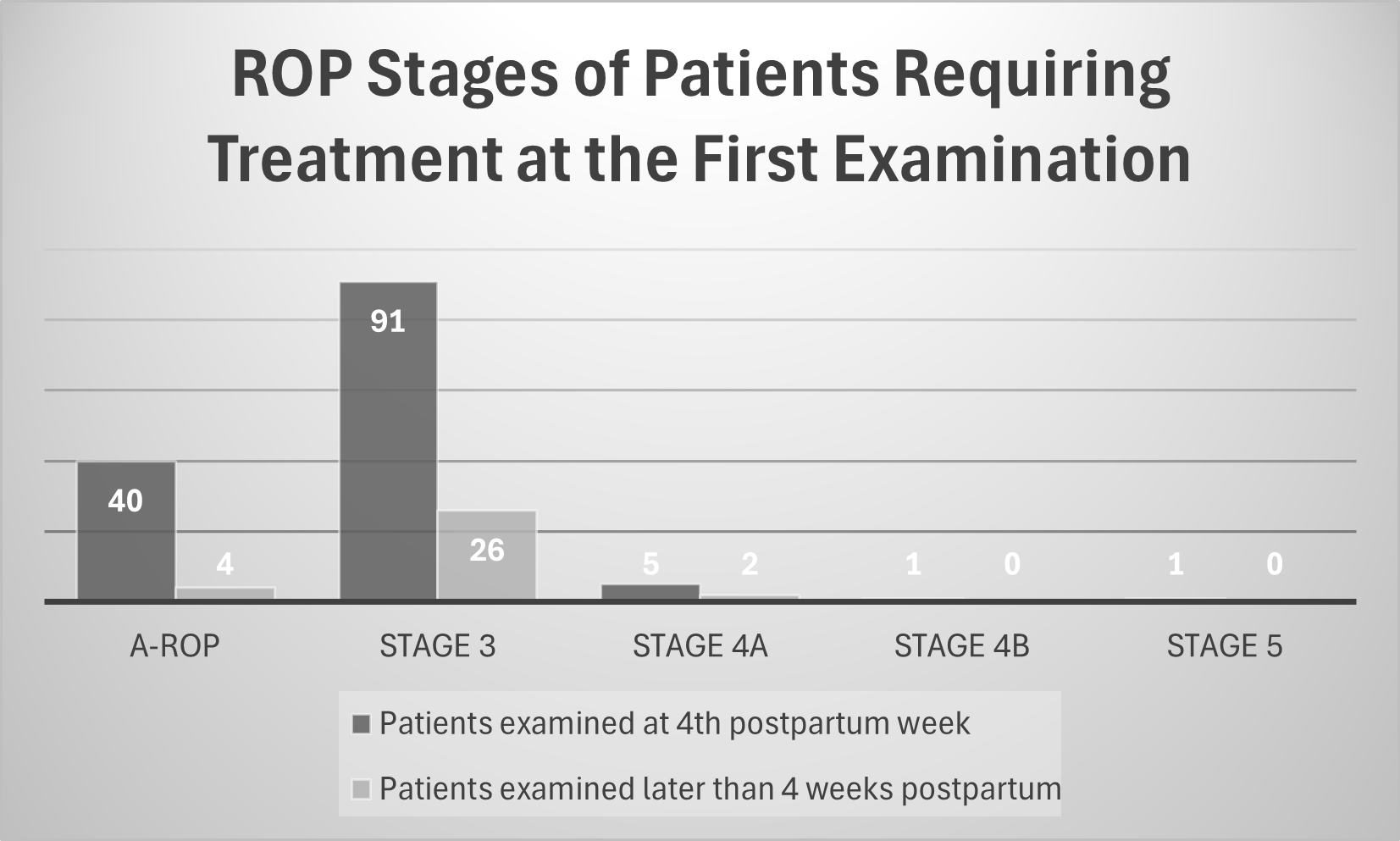

Supplement: online supplemental file 1 [file bmjophth-10-1-s001.tif]
